# Proteomics and multivariate modelling reveal sex-specific alterations in distinct regions of human carotid atheroma

**DOI:** 10.1186/s13293-018-0217-3

**Published:** 2018-12-29

**Authors:** Liam J. Ward, Patrik Olausson, Wei Li, Xi-Ming Yuan

**Affiliations:** 1 Obstetrics and Gynaecology, Department of Clinical and Experimental Medicine, Linköping University, SE-581 85 Linköping, Sweden; 2 Occupational and Environmental Medicine, Department of Clinical and Experimental Medicine, Linköping University, Linköping, Sweden; 3 Pain and Rehabilitation Centre, Department of Medical and Health Sciences, Linköping University, Linköping, Sweden

**Keywords:** Afamin, Atherosclerosis, Lysozyme C, Mass spectrometry, Serine protease inhibitors, Vulnerability

## Abstract

**Background:**

Atherosclerotic lesions are comprised of distinct regions with different proteomic profiles. Men and women develop differences in lesion phenotype, with lesions from women generally being more stable and less prone to rupture. We aimed to investigate the differences in proteomic profiles between sexes, including distinct lesion regions, to identify altered proteins that contribute to these differences observed clinically.

**Methods:**

Carotid endarterectomy samples (ten men/ten women) were obtained, and intraplaque biopsies from three distinct regions (internal control, fatty streak and plaque) were analysed by tandem-mass spectrometry. Multivariate statistical modelling, using orthogonal partial least square-discriminant analysis, was used to discriminate the proteomes between men and women.

**Results:**

Multivariate discriminant modelling revealed proteins from 16 functional groups that displayed sex-specific associations. Additional statistics revealed ten proteins that display region-specific alterations when comparing sexes, including proteins related to inflammatory response, response to reactive oxygen species, complement activation, transport and blood coagulation. Transport protein afamin and blood coagulation proteins antithrombin-III and coagulation factor XII were significantly increased in plaque region from women. Inflammatory response proteins lysozyme C and phospholipase A2 membrane-associated were significantly increased in plaque region from men. Limitations with this study are the small sample size, limited patient information and lack of complementary histology to control for cell type differences between sexes.

**Conclusions:**

This pilot study, for the first time, utilises a multivariate proteomic approach to investigate sexual dimorphism in human atherosclerotic tissue, and provides an essential proteomic platform for further investigations to help understand sexual dimorphism and plaque vulnerability in atherosclerosis.

**Electronic supplementary material:**

The online version of this article (10.1186/s13293-018-0217-3) contains supplementary material, which is available to authorized users.

## Introduction

Atherosclerosis is a multifaceted chronic disease of the arterial wall that is the major cause of cardiovascular disease, the leading cause of mortality in many countries [1]. The epidemiology of this disease shows distinct differences between men and women, with women developing atherosclerosis later in life than men and rapidly developing post-menopause [2, 3]. Hypotheses explaining the differences in onset of atherosclerosis focus on differences between menopausal changes in women. The onset of menopause is accompanied with the reduction of oestrogen levels. Oestrogen increases the production of nitric oxide, a vasodilator, and possesses antioxidant properties [4, 5]. Higher levels of stored iron, with accumulated iron within the plaque as a modifiable risk factor, have been proposed as a hypothesis explaining why the incidence of heart disease is greater in men and post-menopausal women compared to pre-menopausal women [6, 7]. Additionally, the pathophysiology of atherosclerotic lesions show distinct differences between sexes, in general, with women developing more stable plaques, with less intraplaque haemorrhage and thicker fibrous caps that are less prone to rupture than the lesions from men [8, 9].

Atherosclerotic lesions have a high degree of heterogeneity in relation to their morphology and composition; this heterogeneity can affect the progression of atherosclerosis and clinical outcomes [10]. One such morphological feature that can predict the outcome and severity of plaque rupture is the amount of intraplaque haemorrhage present. In general, it has been observed that atheroma from men have more intraplaque haemorrhage [11, 12]. In our previous study, we designed a unique sampling protocol that reduced the effect of heterogeneity by analysing comparable intra-lesion biopsies from distinct regions within carotid endarterectomy samples [13].

Proteomics, much like the other ‘Omics’ methodologies, has the capabilities of generating large datasets from small numbers of individuals. These types of datasets are a challenge for statistical analysis as they are not suited to traditional univariate statistics, where *p* value correction is used to control for repeated hypothesis testing. The *p* value correction is used to remove potential false-positives; however, it does so at the cost of removing putative true-positives [14]. Multivariate statistical analysis can account for inter-dependency between biological molecules, by incorporating co-variance between variables [14]. The use of multivariate statistical analysis, using principal component analyses and partial least square modelling, is becoming increasingly popular in helping to interpret such datasets, characterised by low subject-to-variable ratios, produced by proteomic methodologies [14–16].

Previously, our group has used two-dimensional gel electrophoresis and peptide mass fingerprinting methodology to curate distinguishing proteomes for distinct atheroma regions [13]. Herein, analyses were solely based on the comparison of distinct atheroma regions to the respective internal control regions. Moreover, various differences between atheroma regions were found specific to a sex, for example ferritin light chain abundance in the fibrous cap region was higher in men and lower in women when compared to the respective internal control regions [13]. These findings provoke a question whether men differ from women in plaque proteomes. The objective of the current study is to address sex differences across the atheroma proteome, using tandem-mass spectrometry to curate proteomes for men and women, and use multivariate modelling to directly analyse protein abundances between sexes.

This is the first time a multivariate proteomic approach aimed at investigating sexual dimorphism in human carotid atheroma tissue. The aims of this study are to classify the human carotid atheroma for both men and women identifying distinct differences between the proteomes. In addition, we will also aim to identify if any significant differences are specific for distinct atheroma regions. The primary hypothesis of this study is that there will be significant differences in the proteomes of carotid atheroma between men and women, which may help elucidate the differences seen clinically in the development and progression of atherosclerosis. The investigation is a discovery-proteomics study, thus we also aim to stimulate new hypotheses by identifying potential protein candidates for future investigations.

## Materials and methods

### Human carotid samples

Atherosclerotic carotid arteries were obtained from patients, ten men and ten women, included in the Linköping Carotid Study [17]. Clinical characteristics showed no significant differences between sexes in terms of age, statin therapy, diabetes mellitus, hypertension, smoking or stenosis (Table 1). The study was approved by the Linköping University Hospital ethics committee (Linköping, Sweden), and all experiments were performed in accordance to approved guidelines. Written informed consent was obtained from all patients included. Biopsies were sampled from predefined regions in each carotid endarterectomy sample, including internal control, fatty streak and plaque centre, following a method previously established [13]. Thus, three biopsies from each patient were obtained, totalling 60 carotid endarterectomy biopsies, and protein extracted using a previously described method [13]. Briefly, biopsies were snap-frozen in liquid nitrogen, crushed into a fine powder and homogenised in 1 mL TriZol LS reagent (Thermo Fisher Scientific, MA, USA). Protein precipitation using TriZol LS reagent was performed according to manufacturer’s instructions. Precipitated protein pellets were suspended in 500 μL urea sample solution (6 M urea, 2 M thiourea) with 5 μL PefaBloc (Sigma-Aldrich, MO, USA). Protein concentrations was determined by 2D Quant-Kit (Bio-Rad Laboratories, CA, USA), performed to manufacturers guidelines.

**Table 1:** Basic clinical information of male and female patients with carotid atherosclerosis

	Male	Female	*P* value
*n*	10	10	
Age, years ± SEM	73.4 ± 1.9	71.9 ± 2.1	> 0.05
Statin treatment, % (*n*)	70 (7)	80 (8)	> 0.05
Diabetes mellitus, % (*n*)	20 (2)	20 (2)	> 0.05
Hypertension, % (*n*)	90 (9)	90 (9)	> 0.05
Smoking, % (*n*)	10 (1)	30 (3)	> 0.05
Stenosis (%)	88%	73.8%	> 0.05

### Proteomic analysis

Protein extracts were prepared for mass spectrometry analysis as previously described [13]. Separation and analysis of protein samples were performed using liquid chromatography (Easy nLC; Thermo Fisher Scientific) and tandem-mass spectrometry (Orbitrap Velos Pro; Thermo Fisher Scientific). Spectra were processed using MaxQuant v1.5.3 (Max Planck Institute of Biochemistry, Germany) and searched against the UniProt human protein database [18] using 6 ppm mass tolerance for MS scans and 0.5 Da for MS/MS scans; proteins with at least two unique peptides together with a peptide false-discovery rate of less than 1% were considered identified. Modifications included methionine oxidation, cysteine carbamidomethylation and N-terminal acetylation. Protein abundances are presented in terms of label-free quantification (LFQ), which is calculated using the integrated in-software MaxLFQ algorithm available in MaxQuant [19].

### Western blot analysis

Protein extracts were taken from each lesion region and separated on SDS-PAGE gels (gradient 8–16%, Mini-PROTEAN TGX, Bio-Rad Laboratories). The amount of protein extract used for Western blotting analysis differed on optimisation depending on the expression level of the protein; 25 μg of protein was sufficient for the analysis of afamin and antithrombin-III, and 50 μg of protein was required for the analysis of coagulation factor XII. Western blotting was performed as previously described [20]. Primary antibodies used included anti-afamin (dilution 1:500; PA5-29646, Thermo Fisher Scientific), anti-antithrombin-III (dilution 1:1000; PA5-29500, Thermo Fisher Scientific) and anti-coagulation factor XII (dilution 1:1000; AHXII-5155, Haematologic Technologies, VT, USA). Secondary antibodies used were HRP-conjugated goat anti-rabbit for anti-afamin and anti-antithrombin-III primary antibodies, and HRP-conjugated goat anti-mouse for anti-coagulation factor XII primary antibody. Western blots were illuminated using enhanced chemiluminescence solution (GE Healthcare, UK) and visualised using a charge-coupled device camera.

### Statistical analysis

Multivariate discriminant analysis was performed using SIMCA-P+ software v14.0 (Umetrics AB, Sweden). Multivariate modelling was performed by a series of orthogonal partial least squares discriminant analysis (OPLS-DA), in a procedure described previously [21], setting the nominal outcome variable as either men or women. The workflow and model quality evaluation were in accordance to review by Wheelock and Wheelock [14]. Missing values were considered blank, i.e. the protein was not identified in the corresponding sample, and not included within the multivariate statistical modelling. OPLS-DA modelling was used to predict which variables (proteins) were responsible for class (sex) discrimination. There are two values that correspond to the reliability and robustness of an OLPS-DA model; *R*^2^ represents how well the model explains the dataset, and *Q*^2^ is cross-validated and represents the predictive power of the model [14]. Analysis of variance testing of cross-validated predictive residual (CV-ANOVA) was used to test the reliability of the OPLS-DA model; this statistic is run internally within the SIMCA-P+ software package, where a *p* ≤ 0.05 was considered significant. In brief, CV-ANOVA performs a significance test that compares whether the OPLS model has significantly smaller cross-validated predictive residuals than just the variation around the global average. The degree of effect an individual protein has on the OPLS-DA model is represented by the variable influence on projection (VIP) value. In this study, proteins with a VIP ≥ 1.2 with a 95% confidence interval were considered significant. Proteins were grouped by function according to the assigned “Gene Ontology - Biological Process” using information available from the UniProt database [18].

Univariate statistics were performed on those proteins which displayed a VIP ≥ 1.2. Comparisons were performed between men and women, and then specific matched lesion regions; internal control, fatty streak or plaque centre. Data analysed was found to be not normally distributed, determined by Shapiro-Wilk normality tests and all univariate statistical analysis was performed by non-parametric Mann-Whitney *U* test (SPSS v23.0; IBM, UK). Probability values of *p* ≤ 0.05 were considered significant. In parallel, for those proteins that displayed significant differences, a Bonferroni adjustment was performed to produce more conservative probability values.

## Results

Proteomic analysis of lesion biopsies from carotid atheroma revealed the identification of over 1000 unique proteins per biopsy. A full identification list has been presented as a supplement to our previous work by Liang and colleagues [13]; the current tandem-mass spectrometry results were used as validation of previous reported results where only 41 protein identities from the tandem-mass spectrometry were analysed [13]. Herein, in-depth analysis of the complete dataset, including the 950+ proteins that have undergone no previous statistical analyses, was performed using a combination of multivariate and univariate statistical analyses. Analyses resulted in 43 proteins that significantly discriminant lesion samples between men and women, whereby 10 proteins show significant differences between specific matched lesion regions between sexes.

**Fig. 1:**
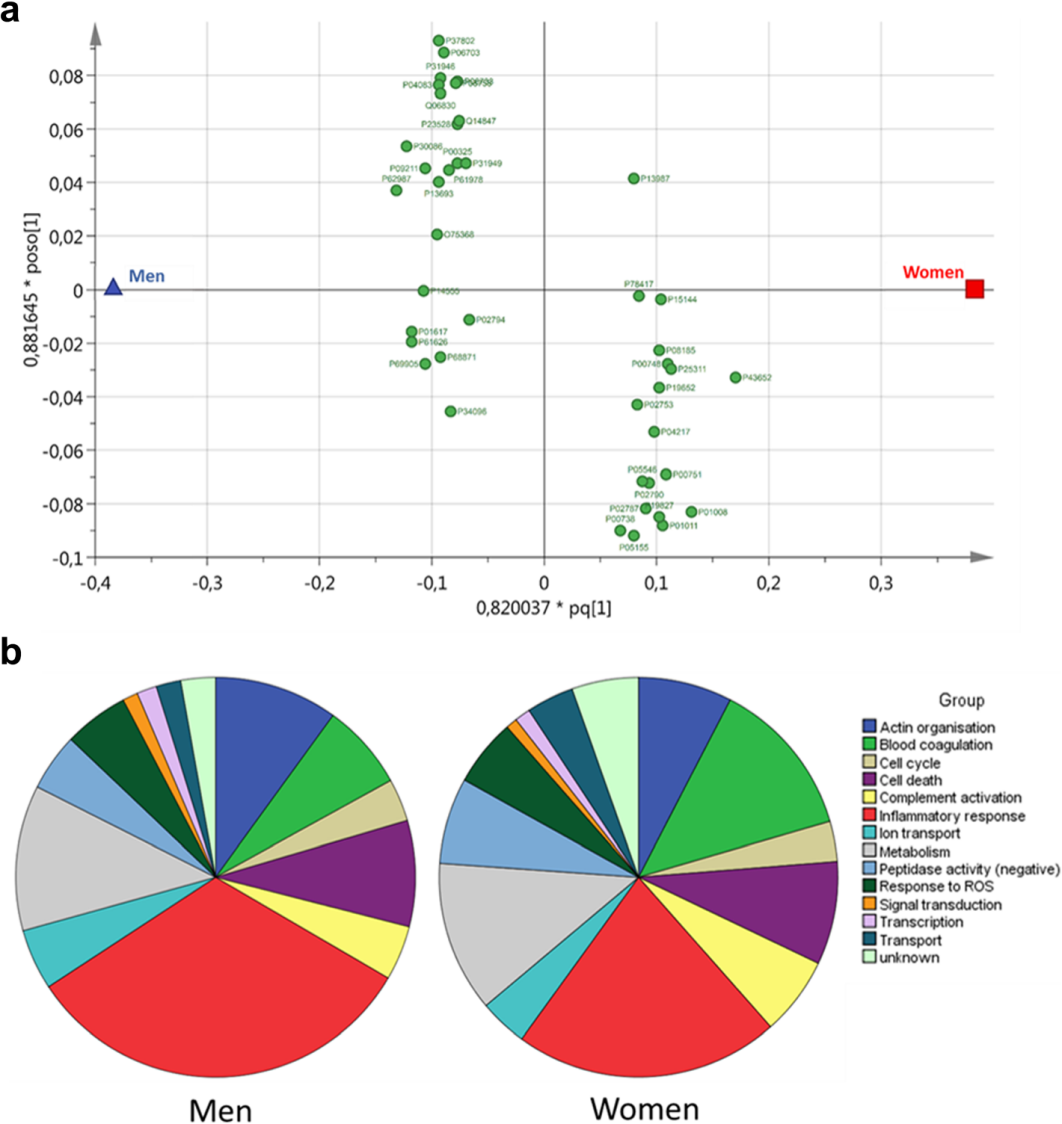
Multivariate modelling reveals that protein alterations within 14 functional groups discriminate carotid atheromas between men and women. Carotid endarterectomy lesions (*n* = 20, equal sex ratio) were sampled from and analysed by tandem-MS. **a** Protein abundances were analysed by multivariate modelling, via orthogonal partial least squares to latent structure discriminate analysis (OPLS-DA), comparing the overall protein abundance between men and women. Loading plot corresponding to proteins with a variable influence on projection (VIP) value > 1.2 were retained, totalling 43 proteins. Resulting model was considered significant with *R*^2^ = 0.83, Q^2^ = 0.49 and a CV-ANOVA of *p* = 0.036. The *x*-axis, pq[1], and *y*-axis, poco[1], depict the predictive component loadings, and the first orthogonal loading, respectively. Proteins to the left of the origin discriminate towards lesion samples from men, and proteins to the right of the origin discriminate towards lesions samples from women. Protein accession numbers correspond to those reported in the UniProt database, and presented in Table 2. **b** Retained proteins from multivariate modelling were grouped by biological process into 14 groups, using information obtained from the UniProt database

Multivariate modelling was performed using LFQ quantification for all identified proteins from the 60 atherosclerotic lesion extracts. An OPLS-DA model with five latent components (one principle and four orthogonal components), with a *R*^2^ = 0.83 and *Q*^2^ = 0.49, and a CV-ANOVA of *p* = 0.036, was constructed. This model indicates significant differences in the protein composition of carotid atheromas between men and women. VIP computations for individual variables reveal that 43 proteins display significant changes (VIP ≥ 1.2), discriminating lesions between men and women (Fig. 1a). Proteins that are further from the origin along the *x*-axis are more discriminant towards the corresponding sex, for example afamin (P43652) is highly discriminant towards lesions from women (Fig. 1a). These discriminating proteins were grouped by biological function into 16 functional groups (Table 2). Results pertaining to functional groups iron homeostasis and haemoglobin/haptoglobin have been previously presented by our group in a parallel study [22]. Figure 1b shows the relative proportions of protein abundances for each functional group for both men and women.

**Table 2:** Proteins identified from human carotid atheroma which discriminate samples by sex, men or women, in accordance with a significant variable influence on projection (VIP ≥ 1.2) in multivariate analysis

Protein name	Accession number	Biological function	VIP	Average LFQ (E + 06)		Fold change men /women	*P* value	Bonferroni adjusted *P* value
				Men mean (SD)	Women mean (SD)			
Cofilin-1	P23528	Actin organisation	1.29	12.4 (12.3)	7.9 (7.2)	1.56	0.270	–
Transgelin-2	P37802	Actin organisation	1.49	26.8 (28.8)	16.8 (17.1)	1.60	0.225	–
Coagulation factor XII	P00748	Blood coagulation	1.51	12.4 (12.3)	7.9 (7.2)	1.56	< *0*.001*	*0*.029*
Antithrombin-III	P01008	Blood coagulation	1.91	14.1 (5.4)	23.3 (10.8)	0.61	< *0*.001*	*0*.018*
Plasma protease C1 inhibitor	P05155	Blood coagulation	1.30	8.3 (6.5)	10.5 (6.4)	0.79	0.138	–
Heparin cofactor 2	P05546	Blood coagulation	1.34	3.9 (3.1)	6.2 (4.2)	0.63	*0*.*014*	0.635
Protein S100-A11	P31949	Cell cycle	1.26	7.9 (5.8)	6.6 (5.9)	1.20	0.266	–
Ubiquitin-60S ribosomal protein L40	P62987	Cell cycle	1.86	5.6 (3.1)	3.9 (2.1)	1.42	*0*.045*	1.950
Annexin A5	P08758	Cell death	1.28	28.3 (18.5)	23.2 (18.7)	1.22	0.163	–
14-3-3 protein beta/alpha	P31946	Cell death	1.43	5.3 (3.5)	4.0 (1.8)	1.33	0.379	–
Complement factor B	P00751	Complement activation	1.66	9.7 (4.2)	14.7 (7.9)	0.69	*0*.025*	1.111
Ig kappa chain V-II region TEW	P01617	Complement activation	1.59	5.7 (2.6)	4.0 (1.8)	1.43	*0*.009*	0.408
CD59 glycoprotein	P13987	Complement activation	1.27	1.9 (1.2)	2.5 (1.9)	0.76	0.237	–
Haemoglobin subunit beta	P68871	Haemoglobin/haptoglobin	1.44	1495 (1426)	1049 (1176)	1.43	0.219	–
Haemoglobin subunit alpha	P69905	Haemoglobin/haptoglobin	1.62	510 (628)	318 (415)	1.61	0.164	–
Haptoglobin	P00738	Haemoglobin/haptoglobin	1.28	117 (151)	161 (170)	0.73	0.054	–
Alpha-1-antichymotrypsin	P01011	Inflammatory response	1.63	17.4 (11.1)	29.1 (20.3)	0.60	*0*.034*	1.453
Annexin A1	P04083	Inflammatory response	1.48	28.3 (13.7)	22.0 (11.5)	1.28	0.065	–
Phospholipase A2, membrane-associated	P14555	Inflammatory response	1.47	2.2 (2.0)	1.1 (1.2)	1.90	*0*.034*	1.478
Alpha-1-acid glycoprotein	P19652	Inflammatory response	1.43	3.9 (2.0)	7.3 (4.3)	0.53	*0*.002*	0.107
Lysozyme C	P61626	Inflammatory response	1.72	74.9 (30.0)	10.2 (9.4)	7.38	*0*.039*	1.686
Protein S100-A6	P06703	Ion transport	1.44	13.2 (15.7)	8.3 (11.9)	1.59	0.093	–
Translationally-controlled tumour protein	P13693	Ion transport	1.35	1.5 (1.0)	1.0 (0.5)	1.51	0.070	–
LIM and SH3 domain protein 1	Q14847	Ion transport	1.23	4.6 (3.0)	3.4 (3.0)	1.37	0.109	–
Serotransferrin	P02787	Iron homeostasis	1.43	179 (61.3)	264 (142)	0.68	*0*.013*	0.559
Haemopexin	P02790	Iron homeostasis	1.49	62.4 (33.7)	104 (73.7)	0.60	*0*.030*	1.28
Ferritin heavy chain	P02794	Iron homeostasis	1.21	54.4 (42.5)	42.9 (38.4)	1.27	0.183	–
Alcohol dehydrogenase 1B	P00325	Metabolism	1.20	15.3 (16.6)	13.0 (26.5)	1.17	0.136	–
Alpha-enolase	P06733	Metabolism	1.30	29.3 (10.1)	23.7 (12.0)	1.23	0.062	–
Aminopeptidase N	P15144	Metabolism	1.41	1.5 (1.1)	2.9 (3.7)	0.53	0.053	–
Inter-alpha-trypsin inhibitor heavy chain H1	P19827	Peptidase activity (negative)	1.62	11.8 (7.3)	18.8 (17.6)	0.63	0.132	–
Phosphatidylethanolamine-binding protein 1	P30086	Peptidase activity (negative)	1.76	6.4 (4.4)	3.7 (2.7)	1.71	*0*.007*	0.286
Glutathione S-transferase P	P09211	Response to ROS	1.58	5.9 (3.2)	4.5 (3.1)	1.31	0.053	–
Glutathione S-transferase omega-1	P78417	Response to ROS	1.20	2.4 (2.4)	3.2 (1.7)	0.75	*0*.009*	0.420
Peroxiredoxin-1	Q06830	Response to ROS	1.42	12.3 (5.8)	10.0 (6.2)	1.23	0.076	–
SH3 domain-binding glutamic acid-rich-like protein	O75368	Signal transduction	1.33	4.8 (6.0)	3.0 (3.0)	1.61	0.178	–
Ribonuclease 4	P34096	Transcription	1.23	4.1 (4.0)	2.7 (1.5)	1.56	0.100	–
Heterogeneous nuclear ribonucleoprotein K	P61978	Transcription	1.24	2.2 (1.2)	1.5 (0.6)	1.45	*0*.030*	1.304
Retinol-binding protein 4	P02753	Transport	1.20	1.8 (0.8)	2.3 (0.9)	0.79	0.051	–
Corticosteroid-binding globulin	P08185	Transport	1.41	0.9 (0.2)	1.4 (0.6)	0.68	*0*.006*	0.275
Zinc-alpha-2-glycoprotein	P25311	Transport	1.63	3.1 (1.1)	4.5 (2.3)	0.70	*0*.041*	1.752
Afamin	P43652	Transport	2.34	2.0 (0.7)	4.4 (2.4)	0.46	< *0*.0001*	< *0*.001*
Alpha-1B-glycoprotein	P04217	Unknown	1.42	11.0 (4.7)	17.6 (9.1)	0.63	*0*.002*	0.077

*significance *p* < 0.05

Univariate statistics were applied, in order to determine if there were significant differences in the abundance per functional group between sexes. Analyses were performed on each functional group that resulted in six functional groups showing significant differences in abundance between sexes; blood coagulation (four proteins; *p* = 0.011), cell cycle (two proteins, *p* = 0.027), ion transport (three proteins; *p* = 0.022), response to reactive oxygen species (ROS, three proteins; *p* = 0.034), transcription (two proteins, *p* = 0.015) and transport proteins (four proteins; *p* < 0.001). In addition, the *unknown* group that only contains alpha-1B-glycoprotein also showed a significant difference between sexes (*p* = 0.002); however, due to the function of this protein being unclear, the biological significance of this result is difficult to infer.

**Fig. 2:**
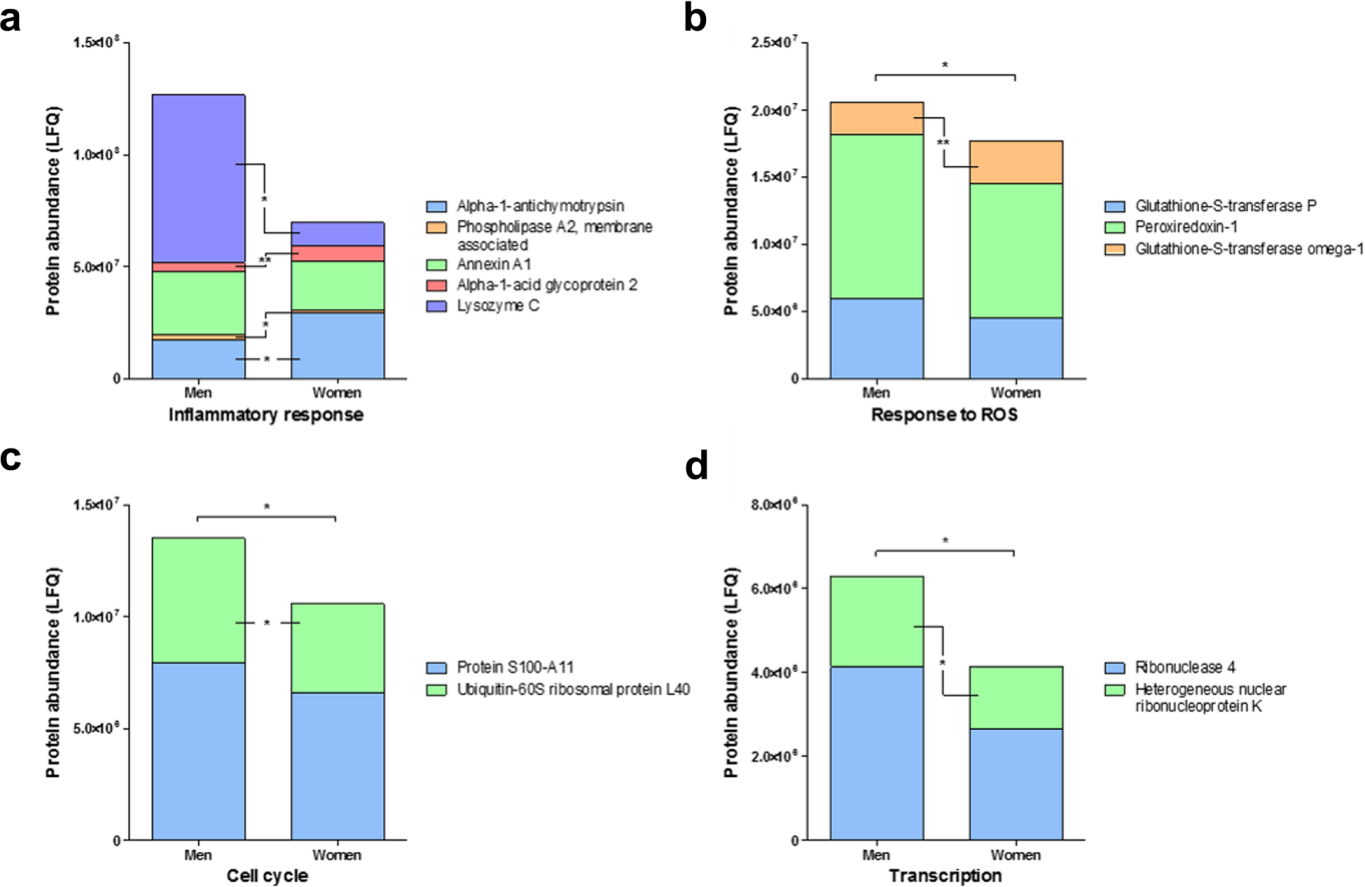
Carotid atheromas from men have a greater protein abundance in four functional groups. Functional groups, as a whole, and then individual proteins belonging to each group were tested for significant differences between men and women via univariate non-parametric Mann-Whitney *U* test, with significant *p* values; p* < 0.05, p** < 0.01 and p*** < 0.001. **a** Inflammatory response, **b** response to reactive oxygen species (ROS), **c** cell cycle and **d** transcription. Individual scatter plots for significantly altered proteins between men and women can be found in Additional file 1: Figure S1

**Fig. 3:**
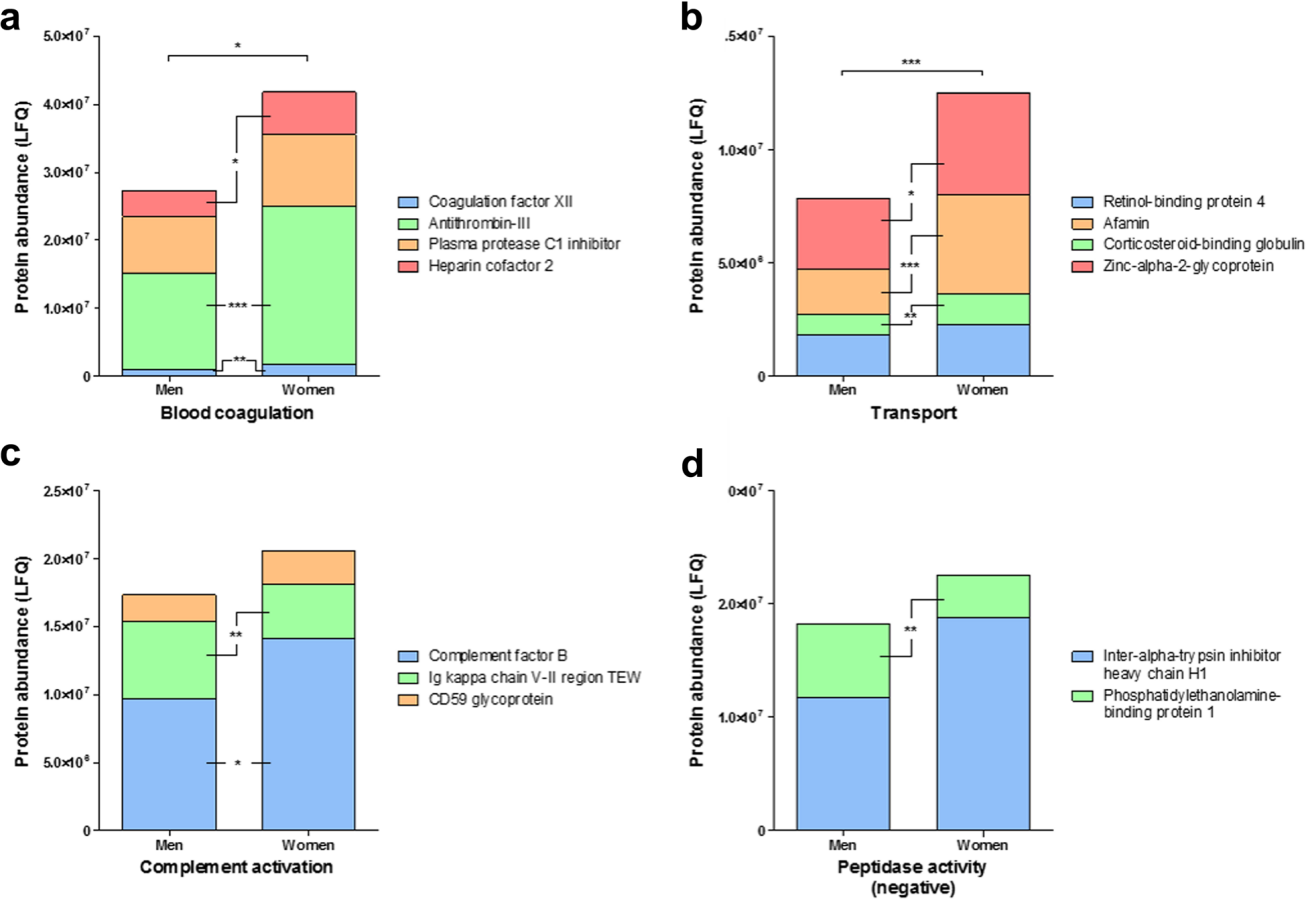
Carotid atheromas from women have a greater protein abundance in four functional groups. Functional groups, as a whole, and then individual proteins belonging to each group were tested for significant differences between men and women via univariate non-parametric Mann-Whitney *U* test, with significant *p* values; p* < 0.05, p** < 0.01 and p*** < 0.001. **a** Blood coagulation, **b** transport, **c** complement activation and **d** peptidase activity (negative). Individual scatter plots for significantly altered proteins between men and women can be found in Additional file 1: Figure S2

In parallel, the differences in abundance per individual protein, within each functional group, was also analysed by univariate analysis (Table 2). In total, 17 proteins, including the aforementioned alpha-1B-glycoprotein, showed significant differences in abundance between men and women (Figs. 2 and 3). In men, inflammatory response proteins showed significantly greater abundance of lysozyme C and phospholipase A2, membrane-associated and significantly less abundance of apha-1-antichymotrypsin and alpha-1-acid glycoprotein 2 in atherosclerotic lesions when compared to women (Fig. 2a). Another three functional protein groups displayed an overall significant increase in abundance in atherosclerotic lesion from men, as compared to women, including response to ROS (Fig. 2b), cell cycle (Fig. 2c) and transcription (Fig. 2d). Specific protein increases, in lesions from men, observed from these groups include ubiquitin-60S ribosomal protein L40 (Fig. 2c) and heterogeneous nuclear ribonucleoprotein K (Fig. 2d). However, glutathione-*S*-transferase omega-1 is significantly higher in women (Fig. 2b). In women, blood coagulation proteins (Fig. 3a) and transport proteins (Fig. 3b) as distinct protein groups display significantly greater abundances in lesions, as compared to men, with specific significant increases in heparin cofactor 2, antithrombin-III and coagulation factor XII (Fig. 3a), and zinc-alpha-2-glycoprotein, afamin and corticosteroid-binding globulin (Fig. 3b). Complement activation proteins showed a significant increase in abundance of complement factor B, and decrease in abundance of Ig kappa chain V-II region TEW in atherosclerotic lesions from women (Fig. 3c). Finally, the group peptidase activity (negative) shows a greater abundance in women, although a significant increase in the abundance of phosphatidylethanolamine-binding protein 1 is present in atherosclerotic lesions from men (Fig. 3d). Individual scatterplots for each protein’s relative abundance can be found in Additional file 1: Figures S1 and S2. A more conservative analysis of the sex differences found was performed via a Bonferroni adjustment of the significant *p* values; this resulted in the retention of significant differences in three proteins, including afamin (*p* < 0.001), antithrombin-III (*p* = 0.018) and coagulation factor XII (*p* = 0.029) (Table 2).

**Fig. 4:**
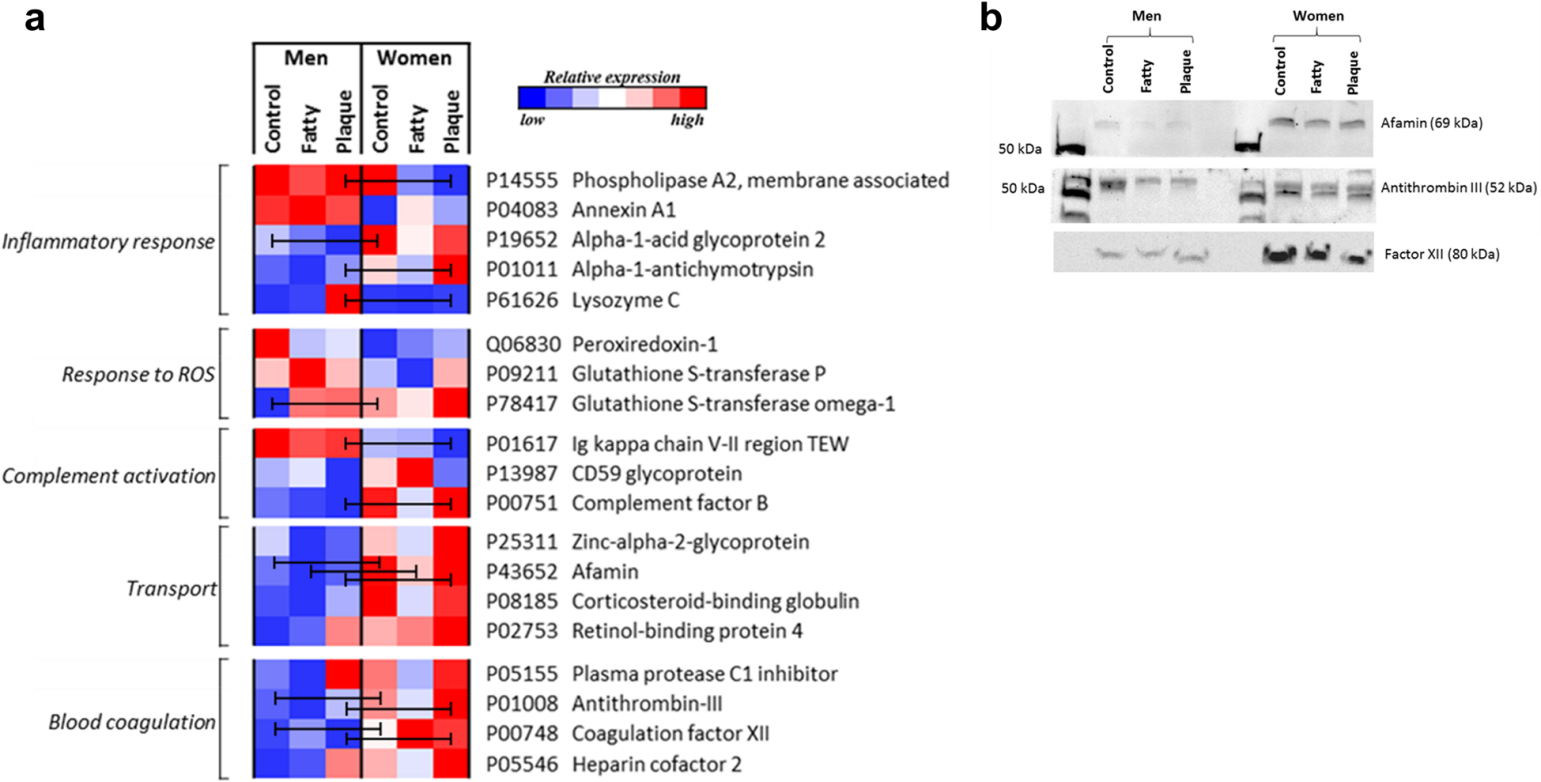
Altered sex-specific proteins show significant differences in matched lesion regions between men and women. Carotid endarterectomy lesions (*n* = 20, equal sex ratio) were sampled isolated specified regions: internal control, fatty streak and plaque centre. **a** Heat map depicting the relative expression of each protein in each lesion region. Colour scale represents a relative shift in protein expression across all lesion regions, men and women, per protein. The lesion region with the highest expression is highlighted in red, through white, and then blue indicating the lesion region with the lowest expression of a protein. Univariate statistical analysis was performed by non-parametric Mann-Whitney *U* test comparing matched lesion regions between men and women, where bars indicate *p* < 0.05. **b** Western blot analysis of altered protein abundances between lesion regions, showing increased abundances of afamin, antithrombin III and coagulation factor XII (Factor XII) in the lesion regions from women. Please note, these are only representations of protein differences in a single man and woman’s carotid endarterectomy samples, and lacks loading control because of technical restrictions and limited amounts of protein samples. Presented gel images are cropped from full-length blots that are presented in Additional file 1: Figure S3

To assess if any discriminating proteins from the multivariate analysis displayed any atherosclerotic lesion region differences, univariate analysis was completed comparing matched lesion regions between sexes; internal control, fatty streak and plaque centre. In total, ten proteins were found to show specific lesion region differences in abundances between men and women (Fig. 4a). The majority of lesion region differences was apparent within the plaque centre regions of lesions from men and women, including significant changes in eight proteins; phospholipase A2 membrane-associated, alpha-1-antichymotrypsin, lysozyme C, Ig kappa chain V-II region TEW, complement factor B, afamin, antithrombin-III and coagulation factor XII. In addition, three proteins were significantly increased in abundance in multiple lesions regions in women, compared to men, including afamin (internal control *p* = 0.030, fatty streak *p* = 0.025, plaque centre *p* = 0.003), antithrombin III (internal control *p* = 0.014, plaque centre *p* = 0.016) and coagulation factor XII (internal control *p* = 0.042, plaque centre *p* = 0.017) (Fig. 4a).

Confirmation of altered protein abundances was performed by Western blot analysis for the three proteins that showed conservative significant differences, after Bonferroni correction, between sexes, including afamin, antithrombin-III and coagulation factor XII. Afamin protein levels were seen greater in all sampled regions from women, compared to men, indicating that women may have greater basal level of afamin (Fig. 4b). Antithrombin-III protein levels were also seen greater in samples from women, and also clearly display the presence of two distinct protein bands, whereas samples from men only show one clear band (Fig. 4b). Coagulation factor XII protein levels were also seen greater in all sampled regions from women compared to men (Fig. 4b). Full-length Western blot images can be found in Additional file 1: Figure S3. Due to the sample source being diseased tissue, conventional house-keeping proteins (beta-actin and GAPDH) were not found to be expressed at stable levels throughout the sample cohort (Additional file 1: Figure S4). Moreover, limited sample availability after proteomic analysis meant that loading controls could not be performed. Thus the Western blotting results are only representative, not conclusive. The overall trend is still in agreement with the primary mass spectrometry analysis showing that women have a greater abundance of afamin, antithrombin-III and coagulation factor XII.

## Discussion

Men and women develop differences in lesion phenotype, with lesions from women generally being more stable and less prone to rupture. However, it is still unclear whether men differ from women in plaque proteomics. In the present study, carotid atherosclerotic lesions were obtained from men and women, where comparative distinct regions of interest were sampled for proteomic analysis by tandem-mass spectrometry together with multivariate and univariate statistical analyses. Previously, a proportion of this tandem-MS dataset, 41 proteins of over 1000 proteins, was used as validation of regional differences identified using two-dimensional gel electrophoresis and peptide mass fingerprinting investigation [13]. Thus, this proportion of the data has been reanalysed for potential sex differences. The analyses resulted in the identification of ten proteins, within five functional groups, that display sex-specific significant alterations in abundance in distinct regions of carotid atherosclerotic lesions (Fig. 4b). Moreover, the multivariate modelling highlighted 43 proteins which significantly discriminate the proteomes of carotid atherosclerotic lesion between men and women, which were grouped by function (Fig. 1a, b).

Atherosclerosis is characterised as an inflammatory disease thus a proportion of the identified discriminating proteins belonging to the inflammatory response group is unsurprising. The inflammatory response group overall displays a non-significant reduced abundance in women compared to men; however, significant differences are found with individual protein abundances. Histologically, atherosclerotic lesions from men are more associated with higher levels of inflammatory infiltrates which contribute to the differences seen in inflammatory response proteins [9, 22]. Phospholipase A2, membrane-associated (also commonly known as secretory phospholipase A_2_-IIA; sPLA2-IIA) and lysozyme C were both significantly increased in men (Fig. 2a), and more specifically significantly increased in the plaque centre region (Fig. 4a). These proteins are associated with atheromatous diseases, with extracellular sPLA_2_-IIA hydrolysing low-density lipoproteins in the vascular wall, promoting the development of foam cells that in turn further secrete sPLA_2_-IIA [23], and increased lysozyme C secretion by plaque macrophages and foam cells are correlated to plaque severity [24]. Interestingly, both of these proteins have been proposed as biomarkers and effectors for cardiovascular disease, with sPLA_2_-IIA being targeted for therapeutic inhibition via treatment with varespladib, albeit failing during clinical trials [25]. Recently, another phospholipase protein, lipoprotein-PLA2, has been found to display a sex-specific increase in abundance and activity in men, and like sPLA_2_-IIA, lipoprotein-PLA2 also promotes inflammation and plaque instability [26]. Other significantly altered inflammatory response proteins included the acute-phase proteins alpha-1-acid glycoprotein 2 and alpha-1-antichymotrypsin, which were both increased in abundance within lesions from women when compared to men, with specific increased abundances within internal control and plaque centre regions respectively. Alpha-1-acid glycoprotein 2, also known as orosomucoid-2, has been found at increased levels in patients with acute myocardial infarction and has anti-inflammatory properties [27]. Vascular expression of alpha-1-antichymotrypsin has been associated with human vascular disease, both in carotid atherosclerosis and abdominal aortic aneurysm, with local increases seen in atherosclerosis where it has been speculated to aide plaque stability [28]. The significantly decreased abundances of sPLA_2_-IIA and lysozyme C, together with the increases in acute-phase proteins specifically the plaque centre, observed in this study may help contribute towards plaque stability in atherosclerotic lesions in women.

Serine protease inhibitors, like the aforementioned alpha-1-antichymotrypsin (serpin A3), were found to discriminate protein compositions between men and women, having increased abundance in lesions of women. These include blood coagulation proteins; antithrombin-III (serpin C1), plasma protease C1 inhibitor (serpin G1), heparin cofactor 2 (serpin D1) and the transport protein, corticosteroid-binding globulin (serpin A6). Antithrombin-III and heparin cofactor 2 inhibit thrombin and its role in blood coagulation [29], events which occur upon atherosclerotic plaque rupture that can cause occlusion of the vessels and lead to catastrophic events such as myocardial infarction and stroke. It has been shown that with age the levels of antithrombin-III and heparin cofactor 2 decrease, increasing the susceptibility of thrombin generation at sites of atherosclerosis [30]. In agreement with the current study, in a large cohort, women have been shown to have higher levels of antithrombin-III than men [31]. The above may suggest that greater levels of antithrombin-III and heparin cofactor 2 may be aiding stabilisation of the atherosclerotic plaque in women by inhibiting thrombus formation. However, an increased abundance of coagulation factor XII (FXII) is observed from the lesions of women, present in both mass spectrometry and western blot analyses, specifically within the control and plaque centre regions, when compared to men. Recently, FXII has been associated with atherosclerotic lesion formation with a greater inflammatory cytokine expression in a *FXII*^−/−^*ApoE*^−/−^ murine model, which would promote plaque instability [32]. Another study has suggested that FXII ensures the stability of thrombi in the later phases of thrombosis after plaque rupture [33]. Interestingly, Western blot analysis shows the presence of two isoforms of antithrombin-III in the lesions from women, whereas only one is clearly present in men (Fig. 4b). This may be indicative of differences in antithrombin-III activity between the sexes, albeit further investigation is required. These sex differences in the abundances of blood coagulation proteins may have implication in the differences of intraplaque haemorrhage in men and women. Men have a greater incidence of intraplaque haemorrhage, compared to women, which is a strong predictor of future clinical events [11, 12]. Functional studies into the activity and/or interactions of antithrombin-III and FXII would provide insight into whether they provide stability, or instability, to atherosclerotic lesions.

Atherosclerotic lesions from women were also abundant in transport-related proteins. The most significant alteration was found when observing the greater abundance of afamin in women, which was the only protein to show a significant increase in all three lesions regions sampled (Fig. 4a). Confirmation via Western blot analysis also displayed the same pattern (Fig. 4b), suggesting that women may have greater basal levels of afamin within the arterial wall. Plasma levels of afamin, a vitamin-E binding glycoprotein, have been strongly associated with the development of metabolic syndrome in three independent human cohorts [34]. Approximately 13% of plasma afamin is suggested to be lipoprotein-associated, mainly in high-density lipoprotein fractions, and the lipoprotein system is where the majority of vitamin-E transport takes place [35]. It has been hypothesised that afamin may be a negative acute-phase protein, as strong negative correlations with inflammatory biomarker, CRP and IL-6, have been observed [36]. Additionally, atherosclerotic lesions from women also show an increase in transport protein zinc-alpha-2-glycoprotein, an adipokine proposed to exhibit similar anti-inflammatory properties as another adipokine adiponectin [37]. These observations are of particular interest in this study, as the increased abundances of afamin and zinc-alpha-2-glycoprotein seen in atherosclerotic lesions from women may be indicative of a lower inflammatory and/or greater anti-inflammatory profile.

### Limitations

The present study utilises a sampling procedure that has been successfully developed to reduce the effect of heterogeneity when analysing atheroma samples by mass spectrometry [13]. In addition, carotid endarterectomy samples were obtained with an equal sex distribution and no significant difference in age or other clinical characteristics between sexes, and internal control samples were taken to help account for individual patient variations. Future studies can benefit from more comprehensive patient information, including other medications (e.g. anti-thrombotics). The lack of comparable healthy arteries that could be used to control for basal differences in the arterial proteomes between men and women is a limitation of the current study. It must also be noted that heterogeneity is still a limiting factor for analysis as atherosclerotic plaques are highly heterogeneous, the biopsies sampled in this procedure contain different amounts of cell populations, extracellular matrix and lipid content that have an effect on the individual proteomes. Due to the number of patients included in this study, there is the possibility for a high level of cellular heterogeneity between biopsies of the same region. In future studies, the cellular heterogeneity between samples of the same region can be controlled for by increasing the sample number and introducing complementary histology. Complementary histology would also be beneficial in validating the cell type differences between sexes, which have an effect on the overall atheroma proteome. Advancing technologies that can combine histology with mass spectrometry, specifically with the advancements in resolution with mass spectrometry imaging technologies will help further this understanding. Another limitation of the current study is that the atheroma used represents latter stage atherosclerosis development. Thus, whether the protein alterations identified represent a casual or an effect implication to atherosclerosis is unclear; future functional studies of the candidate proteins would be required for clarification. The inclusion of other -OMIC strategies, for example lipidomics, transcriptomics and genomics, will help further develop our understanding into sex differences in atherosclerotic development. Finally, the Western blotting in the current investigation lacks loading controls because of technical restrictions and limited protein samples after proteomic analysis. In summary, this study’s limitations should be addressed in future studies by increasing the patient number, with more comprehensive clinical information, and the inclusion of complementary plaque histology to control for cell type differences between sexes.

## Conclusions

Proteomic analysis combined with multivariate modelling, for the first time, has revealed distinct sexual dimorphism in the proteome of human carotid atherosclerotic lesions. Sex-specific differences were identified both in general levels and lesion region-specific perspective. In this study, men have shown to have greater levels of inflammatory response proteins like lysozyme C and sPLA2, and women to have greater levels of serine protease inhibitors and afamin. These differences in the proteome may be suggestive of women developing plaques with a lower inflammatory profile, and greater stability than men. This study has a small sample number and should be considered a pilot study. The discovery-proteomics-based approach, being hypothesis-generating, requires further functional analyses to determine causal/effect relations of the observed proteomic differences. Overall, this study presents a potential proteomic platform for further investigations into the sex differences and plaque vulnerability in atherosclerosis.

## Additional file

Additional file 1: **Figure S1.** Individual scatter plots (mean ± SD) for those proteins that show significant differences in abundance between men and women, displayed in main manuscript Fig. 2. (a) alpha-1-antichymotrypsin, (b) phospholipase A2, membrane associated, (c) alpha-1-acid glycoprotein 2, (d) lysozyme C, (e) glutathione-S-transferase omega-1, (f) ubiquitin-60S ribosomal protein L40, (g) heterogeneous nuclear ribonucleoprotein K. **Figure S2.** Individual scatter plots (mean ± SD) for those proteins that show significant differences in abundance between men and women, displayed in main manuscript Fig. 3. (a) heparin cofactor 2, (b) antithrombin-III, (c) coagulation factor XII, (d) zinc-alpha-2-glycoprotein, (e) corticosteroid-binding globulin, (f) afamin, (g) complement factor B, (h) Ig kappa chain V-II region TEW, (i) phosphatidylethanolamine-binding protein 1. **Figure S3.** Full-length western blot images of the analyses of (a) afamin, (b) antithrombin-III, and (c) coagulation factor XII abundances in atherosclerotic lesions regions from mean and women. Note in blot image (a) the PVDF membrane was cut below 50 kDa for the probing of another primary antibody, hence the differences in contrast, though this test was unsuccessful. **Figure S4.** Protein abundances of (a) beta-actin and (b) GAPDH across the study sample cohort. Both proteins were tested for use as house-keeping protein for quality control of western blot experiments, however due to the unstable abundance across the study sample cohort adequate quality control could not be performed. (PDF 645 kb)
