# Supplementary material for: Proteomics and multivariate modelling reveal sex-specific alterations in distinct regions of human carotid atheroma
Source: Biol Sex Differ. 2018 Dec 29;9:54. doi: 10.1186/s13293-018-0217-3 (PMC6311011; doi:10.1186/s13293-018-0217-3)
Supplement: Supplementary file 1 — Figure S1. Individual scatter plots (mean ± SD) for those proteins that show significant differences in abundance between men and women, displayed in main manuscript Fig. 2. (a) alpha-1-antichymotrypsin, (b) phospholipase A2, membrane associated, (c) alpha-1-acid glycoprotein 2, (d) lysozyme C, (e) glutathione-S-transferase omega-1, (f) ubiquitin-60S ribosomal protein L40, (g) heterogeneous nuclear ribonucleoprotein K. Figure S2. Individual scatter plots (mean ± SD) for those proteins that show significant differences in abundance between men and women, displayed in main manuscript Fig. 3. (a) heparin cofactor 2, (b) antithrombin-III, (c) coagulation factor XII, (d) zinc-alpha-2-glycoprotein, (e) corticosteroid-binding globulin, (f) afamin, (g) complement factor B, (h) Ig kappa chain V-II region TEW, (i) phosphatidylethanolamine-binding protein 1. Figure S3. Full-length western blot images of the analyses of (a) afamin, (b) antithrombin-III, and (c) coagulation factor XII abundances in atherosclerotic lesions regions from mean and women. Note in blot image (a) the PVDF membrane was cut below 50 kDa for the probing of another primary antibody, hence the differences in contrast, though this test was unsuccessful. Figure S4. Protein abundances of (a) beta-actin and (b) GAPDH across the study sample cohort. Both proteins were tested for use as house-keeping protein for quality control of western blot experiments, however due to the unstable abundance across the study sample cohort adequate quality control could not be performed. (PDF 645 kb) [file 13293_2018_217_MOESM1_ESM.pdf]

Supplementary Information

Proteomics and multivariate modelling reveal sex-specific alterations in distinct regions of human carotid atheroma

Liam J. Ward[a,b*], Patrik Olausson[c], Wei Li[a], Xi-Ming Yuan[b].

[a]Obstetrics and Gynaecology, Department of Clinical and Experimental Medicine, Linköping University, Linköping, Sweden.

[b]Occupational and Environmental Medicine, Department of Clinical and Experimental Medicine, Linköping University, Linköping, Sweden.

[c]Pain and Rehabilitation Centre, Department of Medical and Health Sciences, Linköping University, Linköping, Sweden.

*Corresponding author:

Liam J. Ward, MSc. Department of Clinical and Experimental Medicine, Linköping University, SE-581 85 Linköping, Sweden, +46-10-1032478, E-mail: liam.ward@liu.se

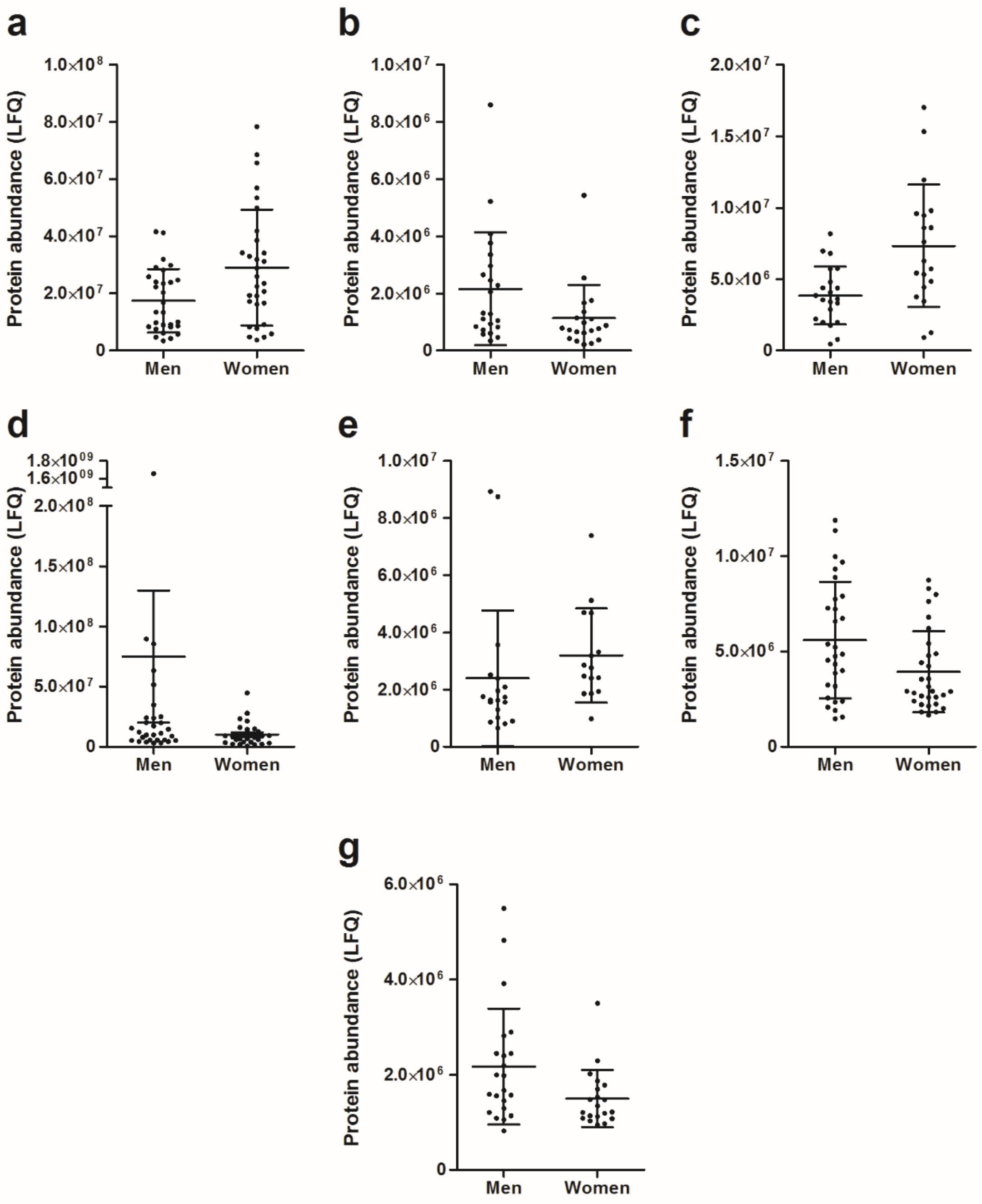

Supplementary Figure S1. Individual scatter plots (mean ± SD) for those proteins that show significant differences in abundance between men and women, displayed in main manuscript Figure 2. **(a)** alpha-1-antichymotrypsin, **(b)** phospholipase A2, membrane associated, **(c)** alpha-1-acid glycoprotein 2, **(d)** lysozyme C, **(e)** glutathione-S-transferase omega-1, **(f)** ubiquitin-60S ribosomal protein L40, **(g)** heterogeneous nuclear ribonucleoprotein K.

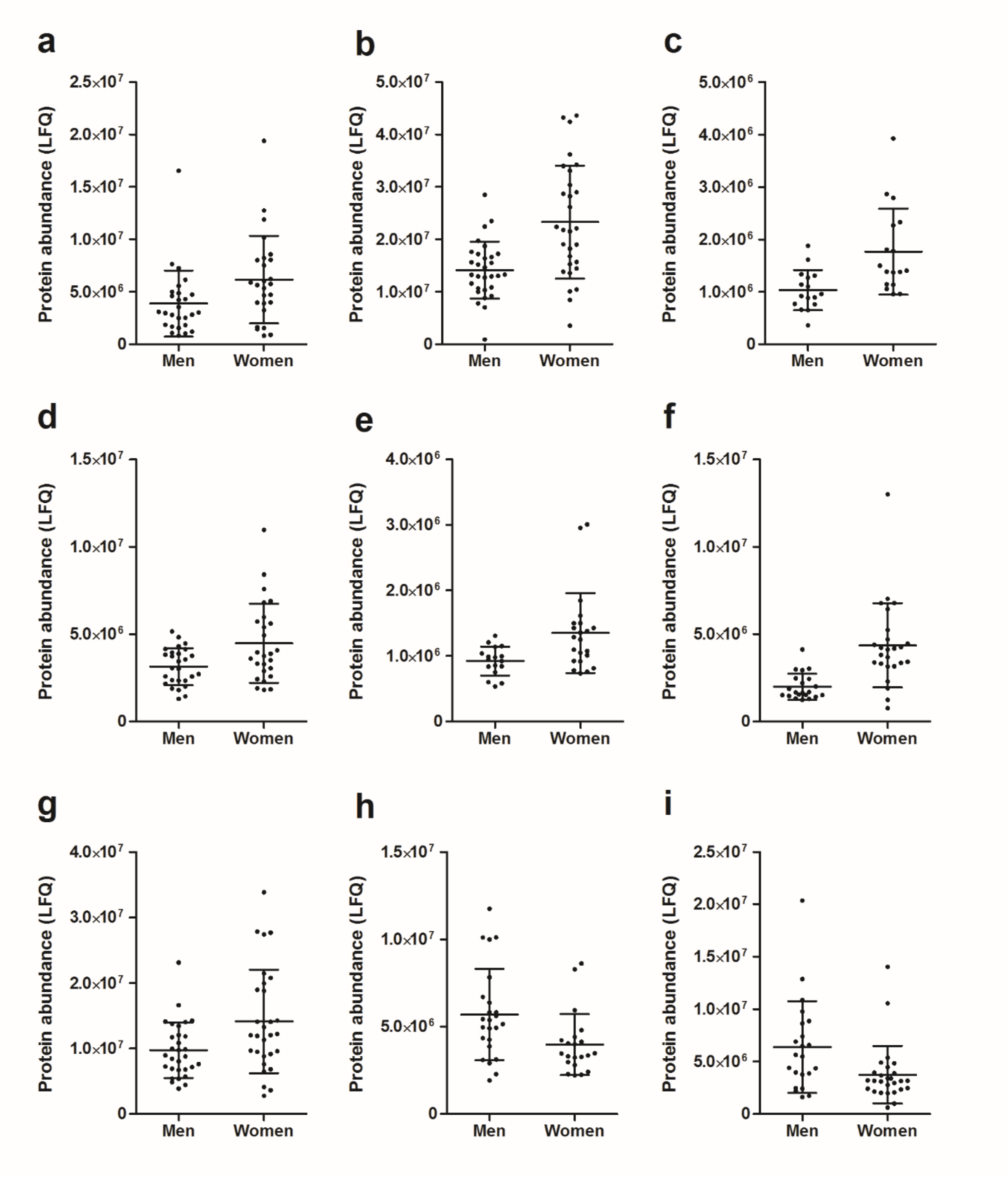

Supplementary Figure S2. Individual scatter plots (mean ± SD) for those proteins that show significant differences in abundance between men and women, displayed in main manuscript Figure 3. **(a)** heparin cofactor 2, **(b)** antithrombin-III, **(c)** coagulation factor XII, **(d)** zinc-alpha-2-glycoprotein, **(e)** corticosteroid-binding globulin, **(f)** afamin, **(g)** complement factor B, **(h)** Ig kappa chain V-II region TEW, **(i)** phosphatidylethanolamine-binding protein 1.

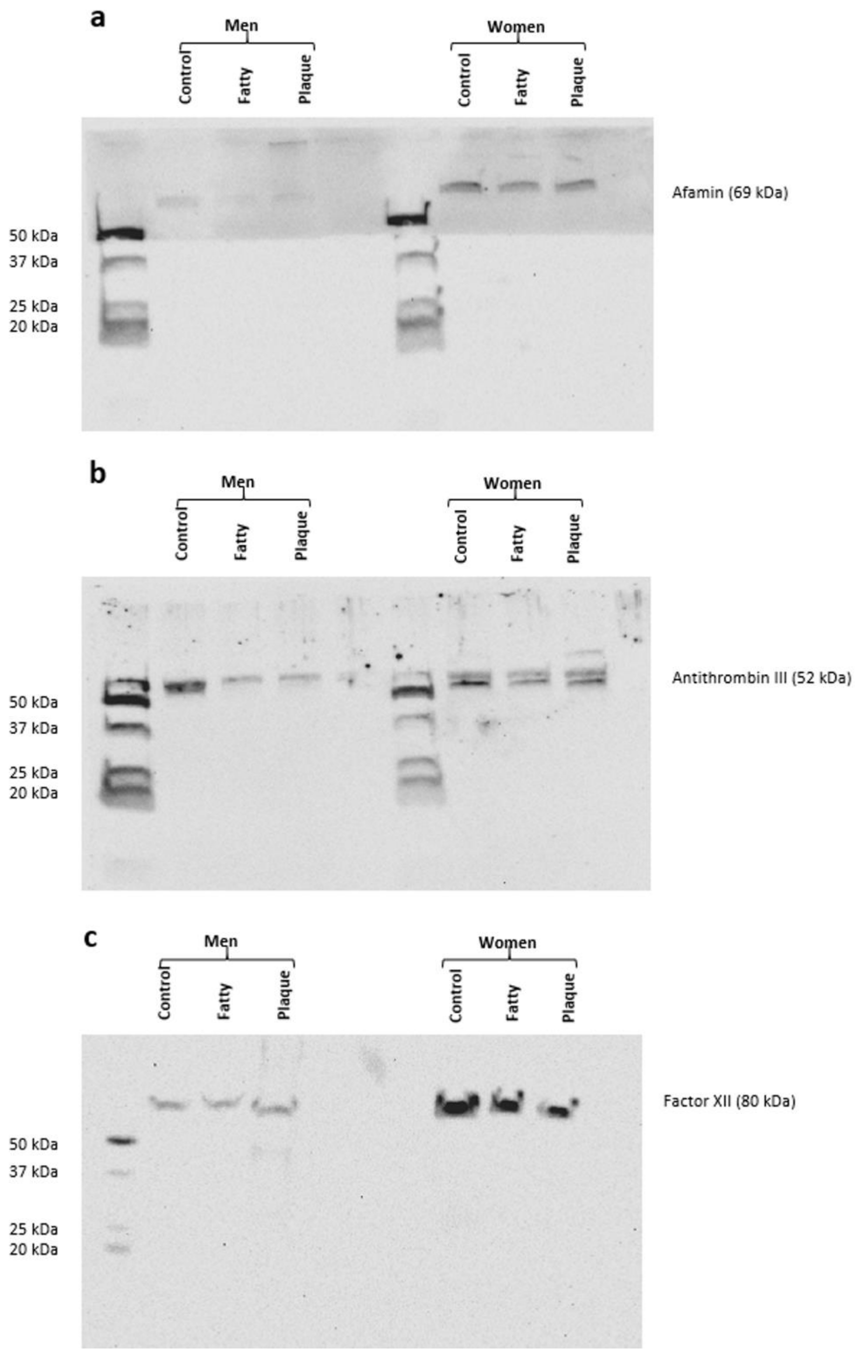

Supplementary Figure S3. Full-length western blot images of the analyses of **(a)** afamin, **(b)** antithrombin-III, and **(c)** coagulation factor XII abundances in atherosclerotic lesions regions from mean and women. Note in blot image **(a)** the PVDF membrane was cut below 50 kDa for the probing of another primary antibody, hence the differences in contrast, though this test was unsuccessful.

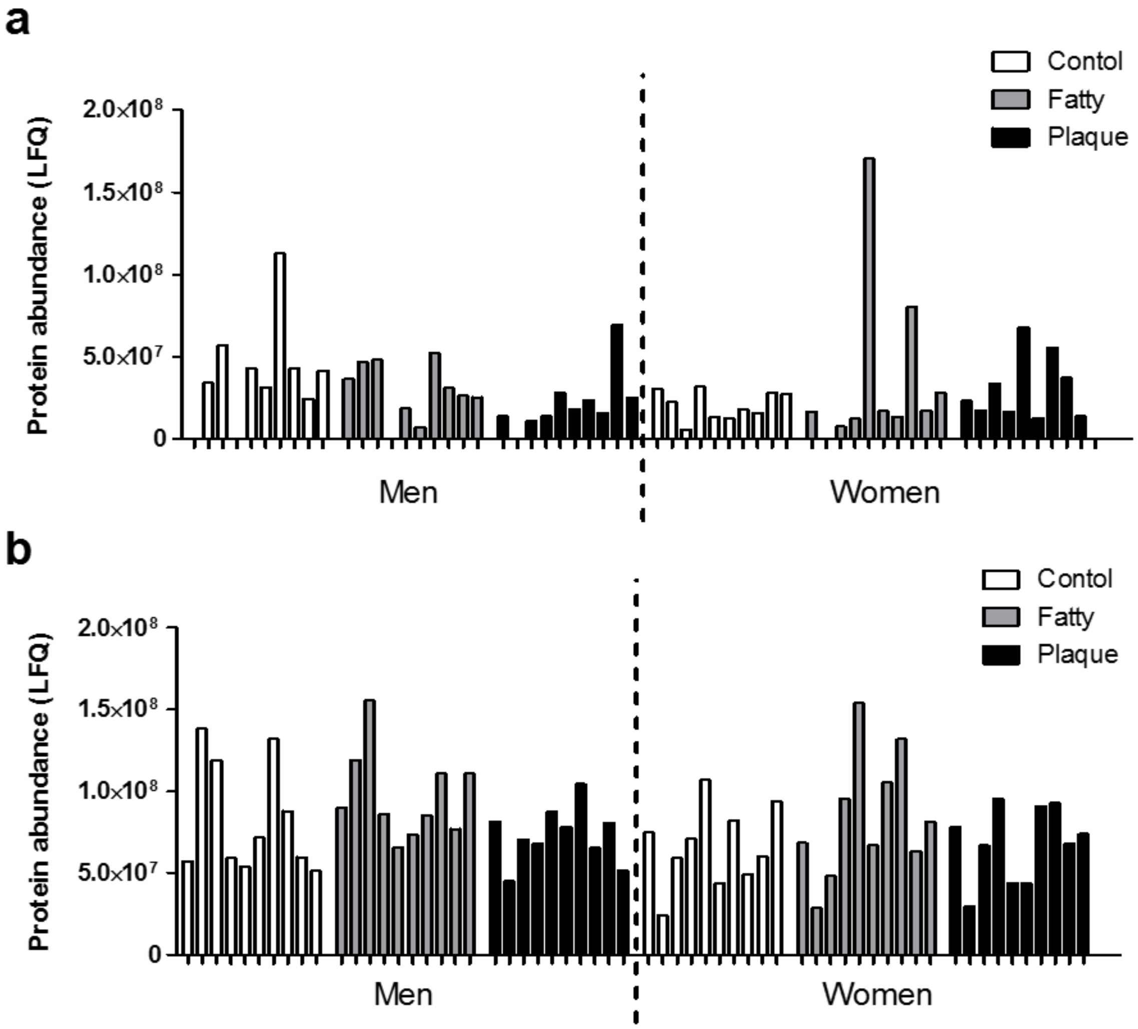

Supplementary Figure S4. Protein abundances of **(a)** beta-actin and **(b)** GAPDH across the study sample cohort. Both proteins were tested for use as house-keeping protein for quality control of western blot experiments, however due to the unstable abundance across the study sample cohort adequate quality control could not be performed.
